# Screening for Peripheral Vascular Disease: The Role of Ankle-Brachial Index (ABI) and Estimated Glomerular Filtration Rate (eGFR) Changes in Chronic Kidney Disease

**DOI:** 10.7759/cureus.71151

**Published:** 2024-10-09

**Authors:** Abinaya Srinivasa Rangan, Nithesh Babu Ramesh, Shreenidhi Rangarajan, Prasanna Karthik Suthakaran

**Affiliations:** 1 Internal Medicine, Saveetha Medical College and Hospital, Saveetha Institute of Medical and Technical Sciences, Saveetha University, Chennai, IND

**Keywords:** atherosclerosis, blood pressure (bp), cardiovascular disease, chronic kidney disease (ckd), peripheral arterial diseases, vascular aging

## Abstract

Background

Chronic kidney disease (CKD) is a significant public health issue worldwide, closely linked with cardiovascular events such as atherosclerosis, peripheral arterial disease (PAD), stroke, coronary artery disease (CAD), and heart failure. The ankle-brachial index (ABI) is a simple tool that compares blood pressure in the ankle and arm, helping to detect PAD and assess the risk of cardiovascular events. An increased ABI signifies an increased risk of cardiovascular disease (CVD), whereas a diminished ABI correlates with angiographically verified atherosclerosis along with heightened death rates. Recent research indicates a U-shaped relationship between ABI and mortality, underscoring the necessity for early detection and action to mitigate potential consequences.

Aim

This study aimed to correlate ABI changes with estimated glomerular filtration rate (eGFR) changes in CKD patients to screen for the development of peripheral vascular disease.

Materials and methods

This prospective cross-sectional study was conducted in the outpatient department of a tertiary care hospital in India, focusing on patients diagnosed with CKD admitted to the general medicine department. The inclusion criteria were CKD patients with an eGFR less than 60 ml/min/1.73 m², accompanied by indicators of renal damage such as albuminuria, reduced kidney size on ultrasound, abnormal urinary sediment, or biopsy-proven kidney disease. Patients with peripheral vascular disease, with end-stage renal disease (on dialysis), with limb amputations, with hemodynamic instability, or who declined participation were excluded. eGFR was calculated using the Modification of Diet in Renal Disease (MDRD) equation, and the ABI was measured at baseline, three months, and six months. ABI values below 0.9 indicated peripheral vascular disease, and the study aimed to correlate changes in ABI with eGFR to assess vascular disease development in non-dialyzed CKD patients.

Results

By the end of the third month, a positive correlation was observed between ABI and eGFR (r=0.259), which persisted at the end of the sixth month (r=0.245).

Conclusion

The study concludes that a direct relationship exists between decreasing eGFR and ABI, which increases susceptibility to PAD and CVD. A low ABI is linked to an accelerated decline in eGFR, indicating that systemic atherosclerosis predicts kidney function deterioration.

## Introduction

Chronic kidney disease (CKD) is a significant global public health issue, with a prevalence between 11.9% and 16.2% [1,2]. CKD is significantly related to cardiovascular incidents along with mortality, consisting of accelerated atherosclerosis, peripheral arterial disease (PAD), coronary artery disease (CAD), stroke, and heart failure [3]. Symptoms of PAD, including cold extremities, peripheral numbness, and muscular cramps, are frequently encountered in individuals with CKD, indicating a notable incidence of PAD in this demographic. The ankle-brachial index (ABI), regardless of being elevated or diminished, functions as a dependable indicator of cardiovascular disease (CVD) risk. A low ABI is significantly linked to a corresponding decrease in estimated glomerular filtration rate (eGFR), indicating that systemic atherosclerosis is vital in predicting renal function impairment [4,5]. Recent research has identified a U-shaped association between ABI and mortality, underscoring the significance of ABI in risk evaluation [6]. Screening for peripheral vascular disease is crucial for preventing complications. This research attempts to discover alterations in ABI and relate them with changes in eGFR in CKD patients to screen for the onset of peripheral vascular disease.

## Materials and methods

Study type and site

The research was conducted as a prospective cross-sectional research in the general medicine department of Saveetha Medical College and Hospital located in Chennai, India.

Study population

The study population comprised 100 patients who presented to the general medicine outpatient department with the diagnosis of CKD or were subsequently diagnosed with CKD after evaluation.

Inclusion and exclusion criteria

The inclusion criteria comprised patients diagnosed with CKD presenting with decreased eGFR (<60 ml/min/1.73 m^2^) and one or more markers for kidney damage, including albuminuria (urine albumin creatinine ratio >30 mg/g or >3 mg/mmol), structurally reduced kidney size (<8 cm in ultrasound with an increase in cortical echoes and cognitive motor dissociation (CMD)), urinary sediment abnormalities (microhematuria, renal tubular epithelial cells), or biopsy-proven kidney disease.

Patients with a pre-existing diagnosis of peripheral vascular disease and final-stage renal disease (stage 5 CKD with eGFR <15 ml/min undergoing dialysis), who had amputations of the upper or lower limbs, who were hemodynamically unstable, or who declined to participate were excluded.

Data collection

After obtaining informed consent, baseline investigations were done such as renal function test, ultrasound of the abdomen, and routine urine culture, and data was collected. The eGFR was calculated using the standard Modification of Diet in Renal Disease (MDRD) equation. The MDRD equation used is as follows: GFR=175×serum Cr-1.154×age-0.203×1.212 (if the patient is an African American in racial origin)×0.742 (if female) [7].

The ABI was measured by recording systolic blood pressures at the brachial artery and at the dorsalis pedis artery. Three recordings of the upper limb and lower limb were taken, and ABI was calculated by dividing the highest ankle systolic pressure by the highest brachial systolic pressure. These evaluations were conducted at baseline, three months, and six months. ABI values less than 0.9 indicated peripheral vascular disease. ABI values more than 1.4 indicated arterial stiffness due to calcification. The normal range for ABI was between 0.9 and 1.4 [8]. The study correlated changes in ABI with eGFR over time to assess peripheral vascular disease development in non-dialyzed CKD patients.

Ethical considerations

Saveetha Medical College and Hospital Institutional Ethics Committee in Chennai provided ethical permission for the study (approval number: SAV/AP/22/92). Each participant signed a consent form or provided a thumbprint prior to the completion of the standard structured questionnaire and blood sample collection. All data were anonymized to protect participant confidentiality.

Participants received standard medical care in accordance with the established protocol without any costs to the participants or the hospital. No personal or professional benefits were provided to participants from any commercial organizations.

Statistical analysis

Data were analyzed using IBM SPSS Statistics for Windows, Version 27.0 (Released 2020; IBM Corp., Armonk, New York, United States). Continuous variables such as eGFR and ABI were expressed as means with standard deviations. Categorical variables were presented as frequencies and percentages. Repeated measures ANOVA was used to assess changes in eGFR and ABI over time (months 0, 3, and 6). Mauchly's test for sphericity was performed, and where sphericity was violated, the Greenhouse-Geisser correction was applied. A statistically significant difference in eGFR over time was identified (F=23.039, p<0.001), indicating a progressive decline in kidney function. Similarly, the ABI showed a statistically significant decrease over time (F=37.576, p<0.001). Pearson correlation coefficients were calculated to assess the relationship between eGFR and ABI at the three time points, revealing a positive correlation that strengthened over time, particularly at the third (r=0.486, p<0.01) and sixth months (r=0.494, p<0.01). A p-value of less than 0.05 was considered statistically significant for all analyses.

## Results

Table 1 summarizes the clinical-demographic details of the study participants. Most participants (48%) are aged 41-60 years, with 69% being male and 31% female. Age and gender distributions are shown, with a total of 100 participants. 

**Table 1:** Demographic details of the study population

Characteristic	Category	Number of participants (n)	Percentage (%)
Age range (years)	20-40	20	20%
	41-60	48	48%
	61-80	28	28%
	>80	4	4%
Mean age±SD		54.26±15.7 years	
Total (age)		100	100%
Gender	Male	69	69%
	Female	31	31%
Total (gender)		100	100%

Table 2 presents both the descriptive statistics (mean±SD) for the eGFR at different time points (months 0, 3, and 6) and the results of the repeated measures ANOVA used to analyze changes in eGFR over time. Mauchly's test indicated a violation of sphericity (p=0.000), so the Greenhouse-Geisser correction was applied (ε=0.723). The F-value (23.039) and p-value (p=0.000) suggest a statistically significant difference in eGFR over time.

**Table 2:** Comparing eGFR at different times in non-dialyzed CKD patients eGFR: estimated glomerular filtration rate; CKD: chronic kidney disease

Time point	eGFR (mean±SD) (ml/min/1.73 m²)	Mauchly's W	Significance (p-value)	Greenhouse-Geisser epsilon	F-value	Significance (p-value)
Month 0	35.60±11.64	0.722	0.000	0.723	0.723	0.000
Month 3	34.70±12.00	0.722	0.000	0.723	23.039	0.000
Month 6	34.19±12.20	0.722	0.000	0.723	23.039	0.000

Table 3 provides both the descriptive statistics (mean±SD) for ABI at different time points (months 0, 3, and 6) and the statistical results from the repeated measures ANOVA. Mauchly's test revealed a breach of sphericity (p=0.000), necessitating the application of the Greenhouse-Geisser correction (ε=0.722). The F-value of 0.051 and a significance level of p=0.000 suggest a statistically significant difference in ABI over time.

**Table 3:** Comparison of ABI at different times in non-dialyzed CKD patients ABI: ankle-brachial index; CKD: chronic kidney disease

Time point	ABI (mean±SD)	Mauchly's W	Significance (p-value)	Greenhouse-Geisser epsilon	Type 3 sum of squares	df	Mean square	F-value	Significance (p-value)
Month 0	0.883±0.032	0.338	0.000	0.722	0.061	1.203	37.576	0.051	0.000
Month 3	0.861±0.057	0.338	0.000	0.722	0.061	1.203	37.576	0.051	0.000
Month 6	0.848±0.055	0.338	0.000	0.722	0.061	1.203	37.576	0.051	0.000

Table 4 illustrates the Pearson correlation coefficients between eGFR and ABI at months 0, 3, and 6 in non-dialyzed CKD patients. The analysis reveals significant positive correlations between eGFR and ABI at all time points, with the strongest correlations observed at the third and sixth months. While the correlation between eGFR and ABI at month 0 is weaker (r=0.243, p<0.05), it strengthens over time, reaching moderate levels by month 3 (r=0.486, p<0.01) and month 6 (r=0.494, p<0.01). These findings suggest that as kidney function declines, ABI decreases in a predictable manner, indicating worsening peripheral arterial health.

**Table 4:** Pearson correlation coefficients between eGFR and ABI at months 0, 3, and 6 in non-dialyzed CKD patients *Indicates significance at p<0.05 **Indicates significance at p<0.01 Correlation coefficient (r) interpretation: r=0.00-0.30: weak; r=0.31-0.50: moderate; r=0.51-1.00: strong eGFR: estimated glomerular filtration rate; ABI: ankle-brachial index; CKD: chronic kidney disease

	eGFR month 0 (ml/min/1.73 m²)	eGFR month 3 (ml/min/1.73 m²)	eGFR month 6 (ml/min/1.73 m²)	ABI month 0	ABI month 3	ABI month 6
eGFR month 0	1	0.993**	0.979**	0.243*	0.486**	0.494**
eGFR month 3	0.993**	1	0.984**	0.271**	0.509**	0.514**
eGFR month 6	0.979**	0.984**	1	0.251*	0.495**	0.495**
ABI month 0	0.243*	0.271**	0.251*	1	0.533**	0.525**
ABI month 3	0.486**	0.509**	0.495**	0.533**	1	0.953**
ABI month 6	0.494**	0.514**	0.495**	0.525**	0.953**	1

**Figure 1:**
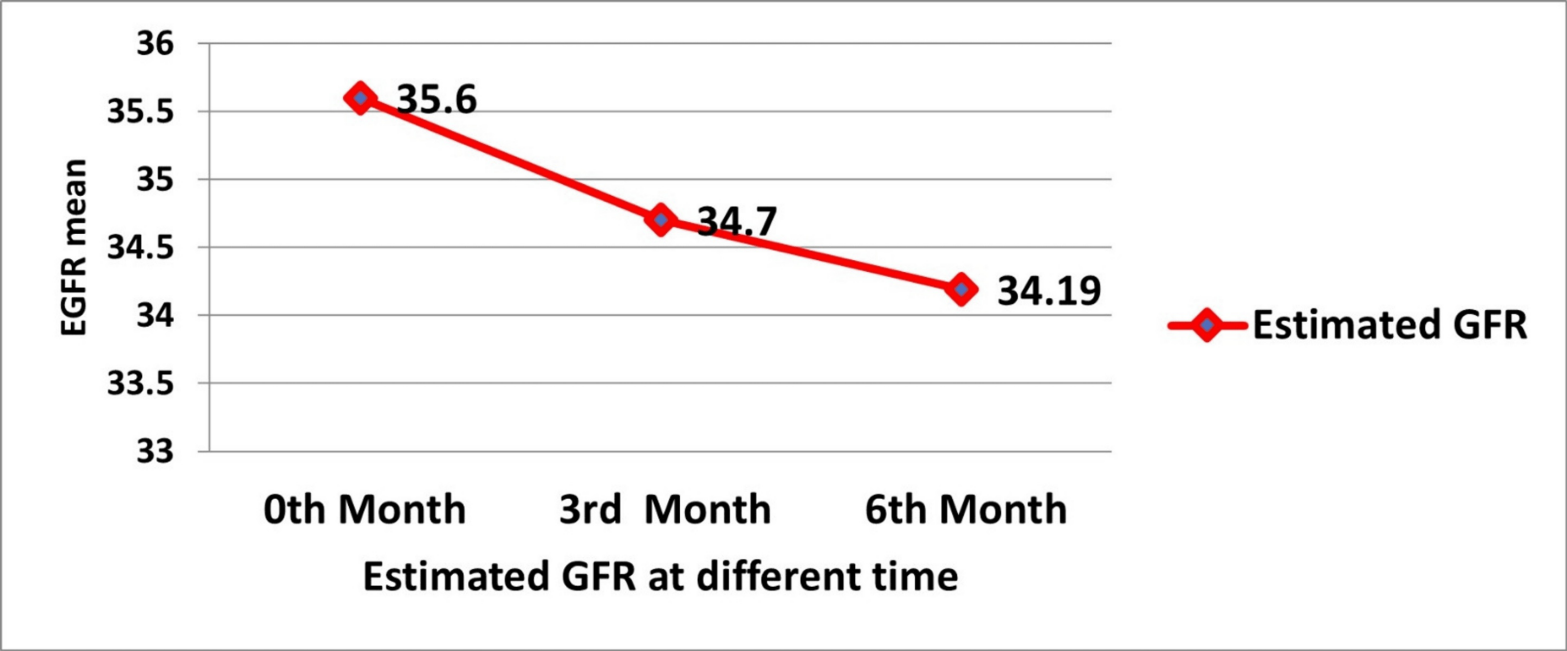
Comparison of eGFR at different times (months 0, 3, and 6) in non-dialyzed CKD patients eGFR: estimated glomerular filtration rate; CKD: chronic kidney disease

Figure 1 illustrates the progressive decrease in eGFR over months 0, 3, and 6 in non-dialyzed CKD patients. A steady decrease in kidney function is observed, with mean eGFR values dropping from 35.60±11.64 ml/min/1.73 m² at baseline to 34.19±12.20 ml/min/1.73 m² at six months.

**Figure 2:**
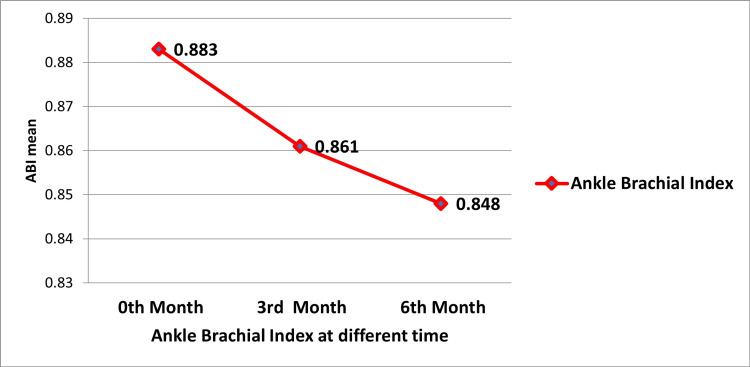
Comparison of ABI at different times (months 0, 3, and 6) in non-dialyzed CKD patients ABI: ankle-brachial index; CKD: chronic kidney disease

Figure 2 shows the decrease in ABI over time in non-dialyzed CKD patients. ABI values decreased significantly from 0.883±0.032 at baseline to 0.848±0.055 at six months, indicating a worsening of peripheral arterial health.

**Figure 3:**
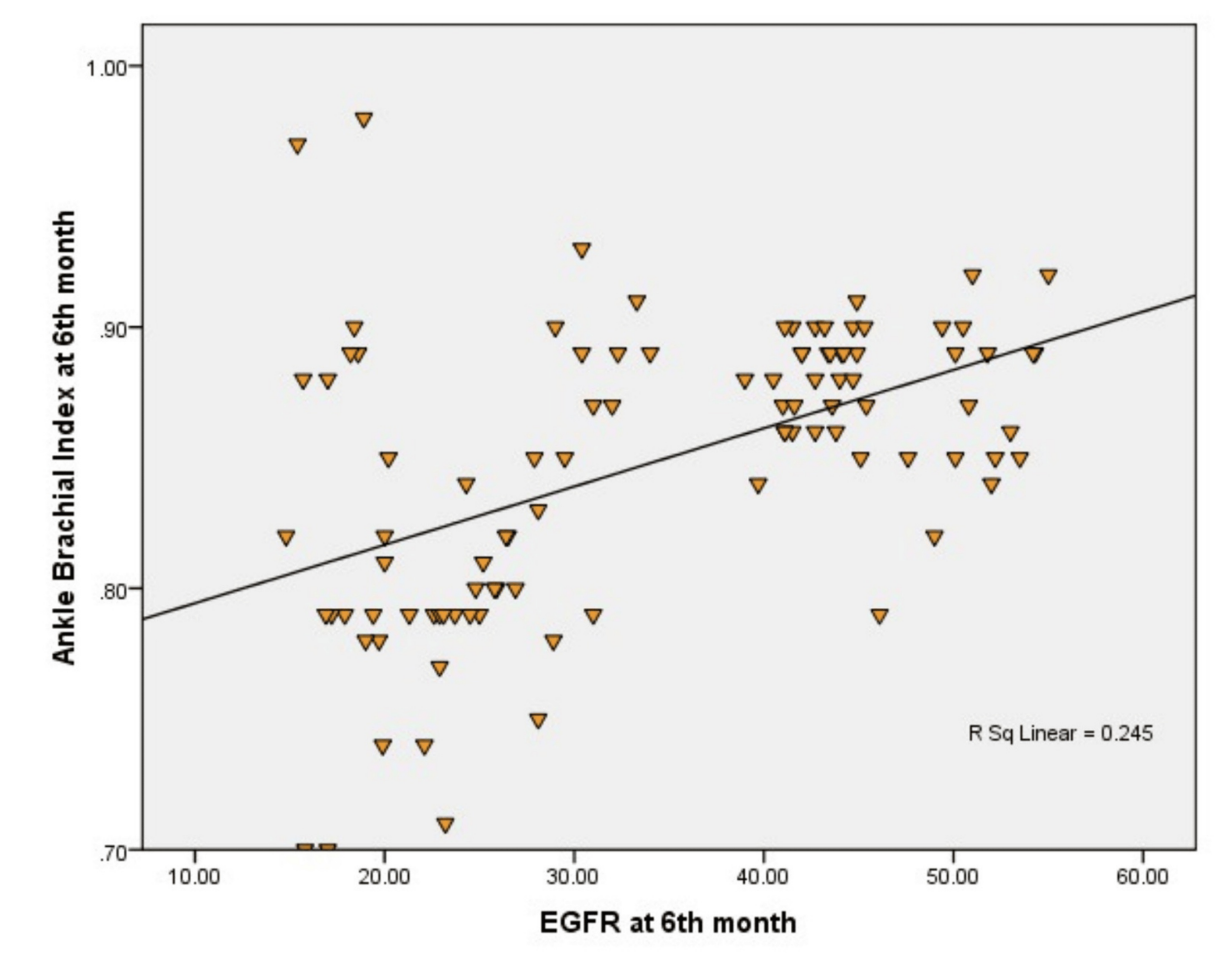
Comparison of eGFR at six months and ABI at three months in non-dialyzed CKD patients by Pearson correlation eGFR: estimated glomerular filtration rate; ABI: ankle-brachial index; CKD: chronic kidney disease

Figure 3 illustrates the moderate positive correlation (r=0.245) between eGFR and ABI in the sixth month in non-dialyzed CKD patients, reflecting the ongoing relationship between kidney function decline and peripheral arterial health.

## Discussion

The study included 100 patients with CKD, with a broad age distribution. Most patients were between the ages of 41 and 60, and the majority (69%) were male. This demographic is consistent with previous studies, which indicate that CKD and related complications are more prevalent in middle-aged and older adults, particularly males. The wide age range and gender representation allow the findings to be somewhat generalized, although the small sample size limits the broader applicability. 

Throughout the study period, we observed a progressive decline in eGFR across the three time points (months 0, 3, and 6). The mean eGFR decreased from 35.60±11.64 ml/min/1.73 m² at baseline to 34.70±12.00 ml/min/1.73 m² at three months and further to 34.19±12.20 ml/min/1.73 m² at six months. This decline was statistically significant (p=0.000). This progressive deterioration of kidney function is consistent with the natural course of CKD and has been corroborated by previous research. Studies such as Obideyi et al. highlight the connection between reduced eGFR and elevated risk of PAD and cardiovascular complications, which was also evident in our findings [9].

ABI measurements followed a similar downward trend, with a statistically significant decrease from baseline to the sixth month. A low ABI is a well-established marker for systemic atherosclerosis and PAD, and our study reinforces this association in CKD patients. As reported by Chen et al., a low ABI is linked to an increased risk of myocardial infarction, CVD, and all-cause mortality, findings that align with our results [10]. The significant reduction in ABI over time reflects worsening peripheral arterial health in these patients.

The correlation analysis between eGFR and ABI at different time points revealed a modest but increasing positive association. Initially weak, this correlation strengthened by the third and sixth months, suggesting that as kidney function declines, arterial health also deteriorates in a predictable manner. This observation is important because it suggests that ABI can serve as a useful non-invasive marker for monitoring vascular health in CKD patients and could potentially predict the rapid decline in kidney function, as supported by studies from Laghari and others [11]. Baber et al. further corroborate this, showing that PAD prevalence is 14.8% in patients with reduced eGFR alone, but increases to 25.4% in those with both reduced eGFR and microalbuminuria [12]. This underscores the link between kidney function deterioration and heightened PAD risk, emphasizing the compounded cardiovascular risks for CKD patients with additional markers like microalbuminuria.

Moreover, Chen et al. demonstrated that patients with an ABI below 0.9 had a 3.3-fold increased risk of cardiovascular events compared to those with a normal ABI [13]. This significant elevation in risk aligns with our findings that a low ABI not only is indicative of worsening peripheral arterial health but also serves as a predictive marker for more rapid kidney function decline and increased cardiovascular risk in CKD patients. These findings emphasize the importance of early detection of ABI abnormalities to prevent cardiovascular complications in this population.

Despite these significant findings, our study had several limitations. The small sample size of 100 participants limits the generalizability of the results, and a larger cohort would provide more robust data. Additionally, the six-month study duration may not have been sufficient to capture the full extent of ABI and eGFR changes, considering the slow progression of CKD.

A critical limitation is the use of ABI in CKD patients. ABI can be inaccurate in patients with CKD due to increased vascular calcification, which leads to vascular stiffness or noncompressible vessels. These calcified vessels can result in falsely elevated ABI values, making it difficult to accurately detect PAD. This limitation underscores the need to utilize more reliable diagnostic tools such as toe pressure and the toe-brachial index (TBI). Unlike the larger vessels measured by ABI, the toe vessels are less affected by calcification, offering a clearer assessment of peripheral arterial health. The inclusion of these measures in future studies could provide a more accurate evaluation of vascular health in CKD patients.

Lastly, the exclusion of patients with end-stage CKD and those on dialysis, along with the lack of tracking specific cardiovascular events, limited our ability to assess the full cardiovascular risks in the CKD population. Future studies should address these gaps to provide a more comprehensive understanding.

## Conclusions

Our study demonstrates a significant relationship between declining eGFR and ABI in CKD patients, with a lower ABI indicating increased risks of PAD and cardiovascular complications. The findings suggest that ABI can serve as a valuable tool for predicting kidney function decline and peripheral arterial health, underscoring the importance of early detection and intervention in this high-risk population.
